# Low-level HIV infection of hepatocytes

**DOI:** 10.1186/1743-422X-9-157

**Published:** 2012-08-09

**Authors:** Ling Kong, Walter Cardona Maya, Maria E Moreno-Fernandez, Gang Ma, Mohamed T Shata, Kenneth E Sherman, Claire Chougnet, Jason T Blackard

**Affiliations:** 1 Division of Digestive Diseases, University of Cincinnati College of Medicine, ML 0595, 231 Albert Sabin Way, Cincinnati, OH 45267, USA; 2 Immunovirology and Reproduction Groups, University of Antioquia, Medellin, Colombia; 3 Division of Molecular Immunology, Cincinnati Children’s Hospital Medical Center, Cincinnati, USA

**Keywords:** HIV, Hepatocyte, Liver, Integration, Huh7.5

## Abstract

**Background:**

There are only limited data on whether HIV infection occurs within the liver; therefore, we explored early and late stages of the HIV life cycle in two hepatocyte cell lines – Huh7.5 and Huh7.5_JFH1_ – as well as in primary human hepatocytes.

**Results:**

Integrated HIV DNA was detected in Huh7.5 and Huh7.5_JFH1_ cells, as well as in primary hepatocytes, and was inhibited by the integrase inhibitor raltegravir in a dose-dependent manner. HIV p24 protein was also detected in cell culture supernatants at days 1, 3, 5, and 7 post-infection and was inhibited by AZT, although levels were modest compared to those in a lymphocyte cell line. Culture supernatants from HIV-infected hepatocytes were capable of infecting a non-hepatic HIV indicator cell line.

**Conclusions:**

These results indicating low-level HIV replication in hepatoctyes *in vitro* complement evidence suggesting that HIV has deleterious effects on the liver *in vivo*.

## Background

HIV infection is associated with a number of hepatic and biliary tract disorders, hepatomegaly, and hepatic steatosis [1-5]. Moreover, liver enzyme elevations are frequent in persons with HIV infection even in the absence of viral hepatitis [6,7]. Additionally, we have recently reported that HIV RNA levels are positively associated with FIB-4 score – a non-invasive serum index of hepatic fibrosis – in HIV mono-infected persons even after controlling for other causes of liver disease, thus supporting a potential association between HIV infection and hepatic fibrosis *in vivo *[8]. *In vivo* data further demonstrate the presence of HIV RNA, proviral DNA, and viral proteins in several hepatic cell types, including hepatocytes (reviewed in [9]). Thus, the effects of HIV itself on the liver deserve careful consideration.

Hepatitis C virus (HCV) – a major cause of chronic liver disease – is a significant public health threat worldwide with ~130 million infected persons [10]. Due to their shared routes of transmission, HIV/HCV co-infection is frequent, and liver disease is a major cause of morbidity and mortality in several HIV-positive cohorts [11-13]. It is well established that HIV co-infection is associated with accelerated liver disease and decreased HCV treatment response rates (reviewed in [14]). Until recently, *in vitro* systems to study the complete HCV life cycle were unavailable. Fortunately, a novel cell culture system – hereafter referred to as Huh7.5_JFH1_ – capable of producing infectious HCV particles now permits examination of the complete life cycle in the presence or absence of other pro-viral or antiviral factors [15-18]. Therefore, given the possibility that HIV infection of hepatocytes could have a profound effect on the liver, we investigated early and late stages of the HIV life cycle in the Huh7.5 and Huh7.5_JFH1_ hepatocyte cell lines, as well as in primary human hepatocytes. Such investigations represent an important first step in improving our understanding of HIV pathogenesis in the liver and characterizing pathways by which HIV interacts with co-morbid conditions such as viral hepatitis and/or alcoholic liver disease.

## Methods

### Cell lines and reagents

The Huh7.5 cell line – generated by curing an HCV replicon-containing cell line with interferon [19] – was provided by Apath LLC (St. Louis, MO). The Huh7.5_JFH1_ cell line – which produces infectious HCV genotype 2a virions – was provided by Dr. Guangxiang Luo [20]. The following reagents were obtained through the AIDS Research and Reference Reagent Program, Division of AIDS, NIAID, NIH: zidovudine (AZT), TZM-bl cells [21] from Drs. John Kappes and Xiaoyun Wu and Tranzyme Inc., plasmids pNL4-3 [22] from Dr. Malcolm Martin and pYK-JRCSF [23] from Drs. Irvin Chen and Yoshio Koyanagi, and raltegravir (catalog #11680) from Merck & Company, Inc. Jurkat and 293 T cells were obtained from ATCC. Primary human hepatocytes were purchased from Diagnostic Hybrids (Athens, OH) or BD Biosciences (San Jose, CA) after isolation from non-transplantable livers perfused with HEPES buffer followed by collagenase treatment. Hepatocytes were dissociated mechanically and filtered to obtain a cell suspension. After non-adherent cells were removed, trypan blue exclusion demonstrated >90% viability.

### Virus preparation

HIV_NL4-3_ (CXCR4-utilizing) and HIV_YK-JRCSF_ (CCR5-utilizing) were prepared by transfection of 1 × 10^6^ 293 T cells per well in a 24-well plate with 1 μg of the appropriate full-length infectious HIV plasmid using the FuGene6 transfection reagent (Roche). Transfected cells were incubated at 37°C for an additional 48–72 hours. Virus-containing supernatants were passed through a 0.20 μm filter to remove cellular debris and precipitated in polyethylene glycol at 4°C. Precipitated virus was then centrifuged at 14,000 g for 20 minutes, resuspended in PBS, and frozen at −80°C until use. The level of p24 protein in cell culture supernatants was determined by p24 ELISA (Perkin-Elmer, Boston,MA; lower limit of detection = 4.3 pg/mL) or by titering on TZM-bl cells. For experiments quantifying integrated HIV DNA, viruses were pre-treated with DNase I at 20 U/mL in 10 mM MgCl_2_ for 1 hour at room temperature to eliminate any cellular DNA carryover from virus production.

### HIV infections

5 × 10^5^ cells (Huh7.5, Huh7.5_JFH1_, or primary hepatocytes) were seeded per well of a 24-well, collagen-coated plate and incubated at 37°C in 5% CO_2_ with infectious HIV at a multiplicity of infection (MOI) of 0.25-1.0 in a volume of ~500 uL per well unless otherwise noted. After 4 hours, viruses were removed by washing cells five times with buffer, and fresh medium was added. Cells and cell culture supernatants were removed at various timepoints post-infection to measure HIV p24 protein levels by ELISA. For Western Blots, 100,000 cells were harvested at days 1, 3, 5, and 7 post-infection and lysed in 100 uL of buffer; 10 uL was loaded per well. HIV p24 was detected using a 1:1000 dilution of a rabbit monoclonal antibody (Ab) from Epitomics (Burlingame, CA; catalog # 1523–1) as the primary Ab and a 1:5000 dilution of a rabbit polyclonal Ab to mouse IgG from Abcam (Cambridge, MA; catalog # 6728) as the secondary Ab. As an additional loading control, glyceraldehyde 3-phosphate dehydrogenase (GAPDH) was detected using a rabbit polyclonal Ab from Santa Cruz Biotechnology (Santa Cruz, CA; catalog #SC-25778) at a 1:1000 dilution.

For preparation of non-infectious HIV with functionally intact envelope glycoproteins, HIV_NL4-3_ was first prepared by transfection of 293 T cells as described above. After filter sterilization, virus was inactivated by adding 250 uM of aldrithiol-2 (AT-2; Sigma-Aldrich) to the filtered supernatants as described elsewhere [24]. AT-2-treated viruses were then incubated with the TZM indicator cell line to confirm that they were not infectious (data not shown), and HIV-1 p24 ELISA was used to quantify virus.

To evaluate the production of infectious virions, supernatants from HIV-infected hepatocytes or Jurkats were collected at day 3 post-infection and titered using TZM-bl cells – a HeLa-derived indicator cell line which expresses the β-galactosidase and luciferase genes under the control of the HIV promoter [25]. 1 × 10^5^ TZM-bl cells were infected with supernatants for two days, washed, and fixed with 1% (v/v) formaldehyde and 0.2% (v/v) glutaraldehyde in PBS. Following several washes with PBS, TZM-bl cells were tested for β-galactosidase activity with a solution of 4 mM potassium ferrocyanide/ferricyanide, 2 mM MgCl_2_, and 0.4 mg/mL 5-bromo-4-chloro-3-indolyl-D-galactopyranoside (X-gal). The number of positive cells per well was then counted.

### Quantification of integrated HIV DNA

After 4 hours of HIV infection, 5 × 10^5^ cells were washed and incubated for an additional 24 hours. For experiments with antiviral drugs, cells were incubated with 1–1000 uM of the integrase inhibitor raltegravir or 100uM of the reverse transcriptase inhibitor AZT before and during HIV infection. 7,500 cells were then suspended in lysis buffer containing 10 mM Tris HCl pH9, 0.1% Tween 20-NP40 and 400 μg/mL Proteinase K (Invitrogen). Cellular lysates containing ~7,500 cells were used to quantify integrated HIV DNA by nested real time PCR as described elsewhere with minor modifications [26,27]. Briefly, the first round amplification used *Alu* sequence specific primers and the HIV-1 long terminal repeat (LTR). This reaction was followed by a second round of amplification with specific primers and a specific labeled probe against the LTR performed in a Light Cycler (Roche). Using the ACH-2 cell line, a line of human T-lymphocytic leukemia that contains a single copy of HIV-1 proviral DNA, the limit of detection was 3 copies of integrated HIV DNA per reaction.

## Results

### Detection of integrated HIV DNA in hepatocytes

**Figure 1:**
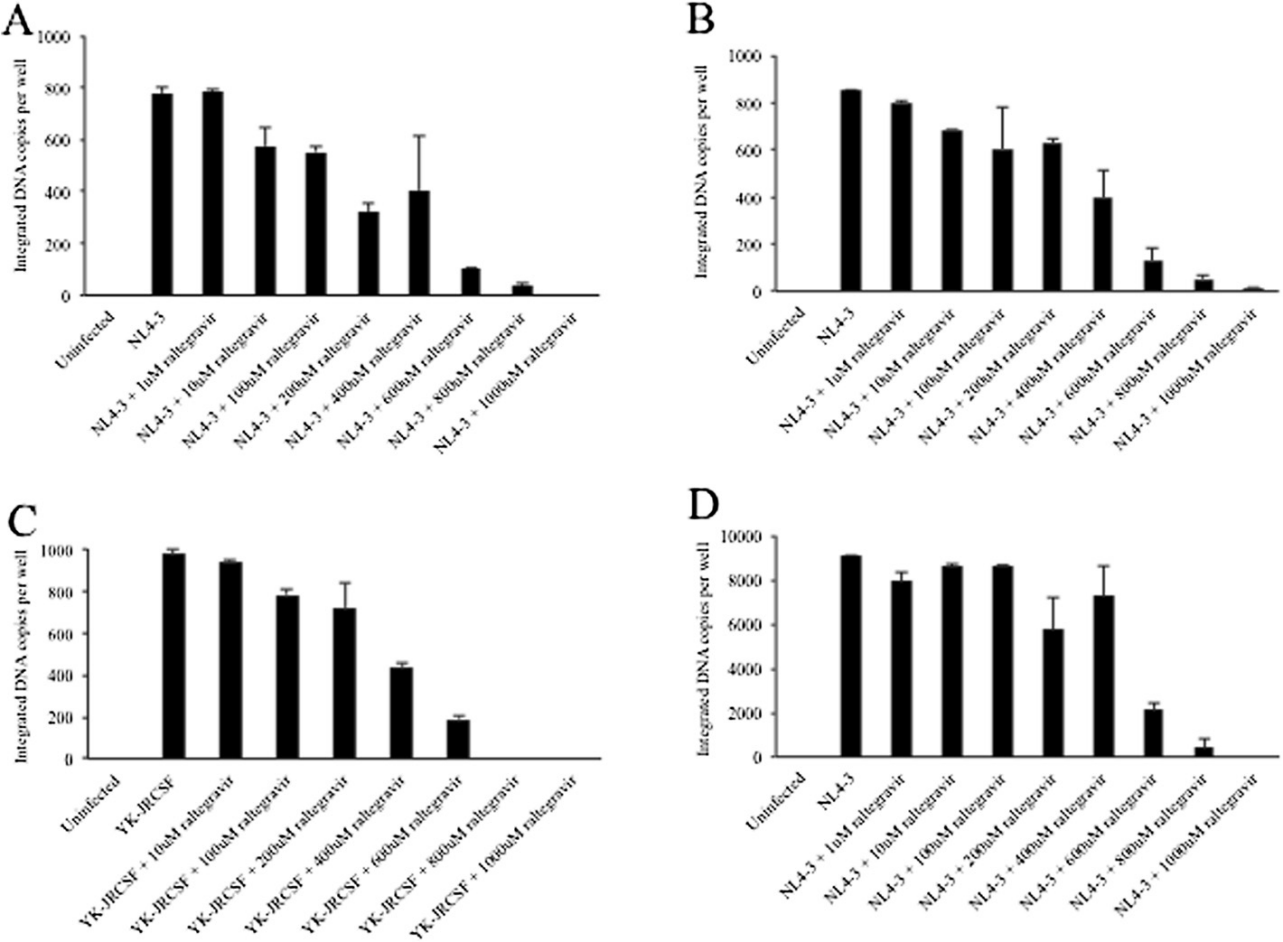
**Detection of integrated HIV DNA in hepatocytes.** Huh7.5 (**A**), Huh7.5_JFH1_(**B**), or Jurkat (**D**) cells were incubated in the presence of 0, 1, 10, 100, or 1000 uM of the integrase inhibitor raltegravir for one hour prior to and during infection with DNase-treated HIV_NL4-3_. Huh7.5 (**C**) cells were incubated in the presence of raltegravir for one hour prior to and during infection with DNase-treated HIV_YK-JRCSF_. After 24 hours, integrated HIV DNA was quantified in cell lysates by nested real-time PCR. Error bars represent the standard deviation between duplicates.

To assess HIV infection of hepatocytes, we first examined whether the Huh7.5 and Huh7.5_JFH1_ cell lines could support early steps in the HIV life cycle. As shown in Figure 1A-B, both hepatocyte cell lines contained integrated HIV DNA after infection with the CXCR4-utilizing lab-adapted strain HIV_NL4-3_ that had been DNase-treated to remove residual input DNA. Addition of the integrase inhibitor raltegravir resulted in a dose-dependent decrease in integrated HIV DNA levels in both cell lines. Infection with the CCR5-utilizing lab-adapted strain HIV_YK-JRCSF_ resulted in similar levels of integrated HIV DNA compared to HIV_NL4-3_ and was also inhibited by raltegravir in a dose-dependent manner (Figure 1C). The Jurkat lymphocyte cell line was evaluated as a positive control, and demonstrated that HIV integration was approximately 10-fold higher in lymphocytes compared to hepatocytes (Figure 1D). In this assay, integrated HIV DNA was regularly tested for in 1) DNase-treated stock virus preparations, 2) washes after removal of residual unbound virus, and 3) mock infected cell lines. In all cases, no integrated HIV DNA was detected, strongly suggesting that these findings were not due to residual input virus.

### Expression of p24 and production of infectious HIV in hepatocytes

**Figure 2:**
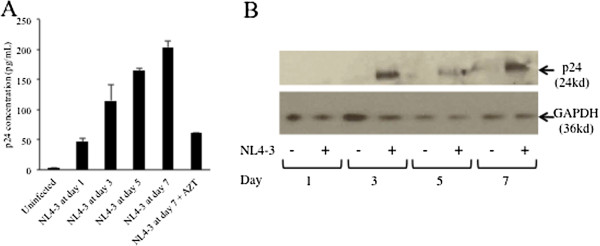
**HIV protein expression in hepatocytes.** (**A**) HIV p24 protein levels were quantified by ELISA in culture supernatants from Huh7.5 cells infected with HIV_NL4-3_ at days 1, 3, 5, and 7 post-infection. Infection was also performed in the presence of 100 uM AZT for one hour prior to and during infection. Error bars represent the standard deviation between duplicates. (**B**) Huh7.5_JFH1_ cells were incubated with HIV_NL4-3_. At days 1, 3, 5, and 7 post-infection, cells were harvested, lysed, and subjected to Western Blot analysis using a rabbit monoclonal Ab (Epitomics) as the primary Ab and a rabbit polyclonal Ab to mouse IgG from (Abcam) as the secondary Ab. As a loading control, GAPDH was detected using a rabbit polyclonal Ab (Santa Cruz Biotechnology).

We next examined whether hepatocytes could also support late stages within the HIV life cycle. Because of the high autofluorescence associated with hepatocytes, flow cytometry was not an optimal choice for these experiments; rather, cumulative HIV p24 antigen expression was quantified in cell culture supernatants collected from Huh7.5 cells infected with HIV_NL4-3_ at day 1 (47.0 ± 4.9 pg/mL), day 3 (115.1 ± 26.8 pg/mL), day 5 (165.7 ± 3.7 pg/mL), and day 7 (203.9 ± 9.8 pg/mL) (Figure 2A). Incubation of Huh7.5 cells before and during infection with 100 uM AZT resulted in a significant decrease in p24 expression (61.4 ± 0.6 pg/mL). These p24 levels in the supernatants of HIV-infected Huh7.5 cells were lower than those observed after HIV_NL4-3_ infection of the Jurkat lymphocyte cell line (data not shown). Additionally, p24 protein expression was detected at days 3, 5, and 7 post-infection via Western Blot in HIV-infected Huh7.5_JFH1_ cell lysates, although this approach was less sensitive than detection via ELISA (Figure 2B).

**Figure 3:**
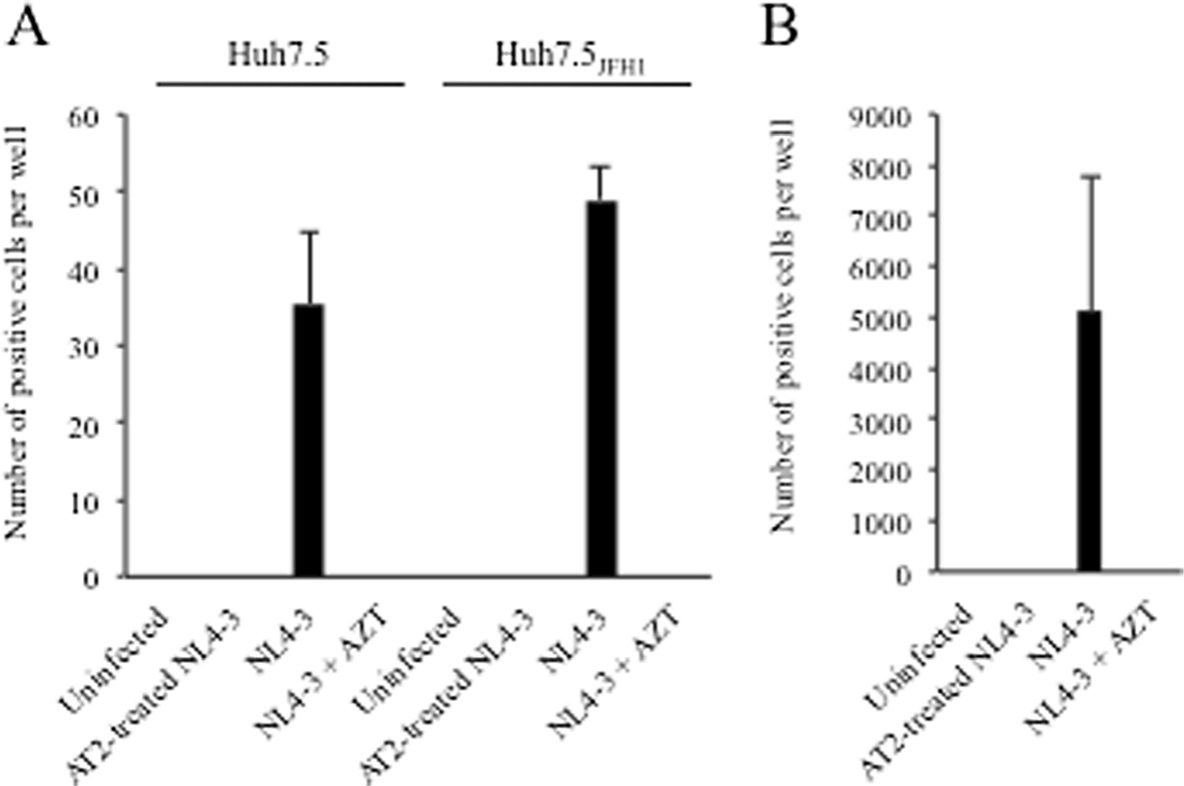
**Infectious HIV production in hepatocytes.** TZM-bl indicator cells were incubated with culture supernatants collected at day 3 post-infection from Huh7.5 or Huh7.5_JHF1_ cells (**A**) or Jurkat cells (**B**) infected with HIV_NL4-3_ or 100 ng AT-treated HIV_NL4-3_and tested for β-galactosidase activity. The mean number of positive TZM-bl cells per well is shown with the error bars representing the standard deviation between duplicates. β-gal expression was also evaluated in the presence of 100 uM AZT for one hour prior to and during infection.

To determine if HIV released from hepatocytes could productively infect cells of non-hepatic origin, the TZM-bl indicator cell line was incubated with supernatants collected on day 3 from HIV-infected hepatocytes. Cell counts were 35.5 ± 9.2 or 49.0 ± 4.2 positive cells per well after incubation with supernatants from Huh7.5 or Huh7.5_JFH1_ cells infected with HIV_NL4-3_, while background values were 0.0 ± 0.0 positive cells for both cell lines treated with the 5^th^ wash of the HIV input or mock infected cells (Figure 3A). Similarly, no positive cells were detected in both cell lines after incubation with 100 ng AT-2-treated HIV_NL4-3_ or 100 uM AZT, suggesting that there was no non-specific activation of the HIV LTR and that TZM-bl positive cells were the result of *bona fide* virion production. Incubation of Huh7.5 or Huh7.5_JFH1_ cells with HIV_YK-JRCSF_-infected supernatants gave similar results, although overall positivity was lower (data not shown). When TZM-bl cells were incubated with supernatants from HIV_NL4-3_-infected Jurkat cells, the number of positive cells was 5125 ± 2651 per well (Figure 3B) suggesting that production of virions is significantly higher in Jurkats than hepatocyte cell lines.

### HIV infection of primary hepatocytes

**Figure 4:**
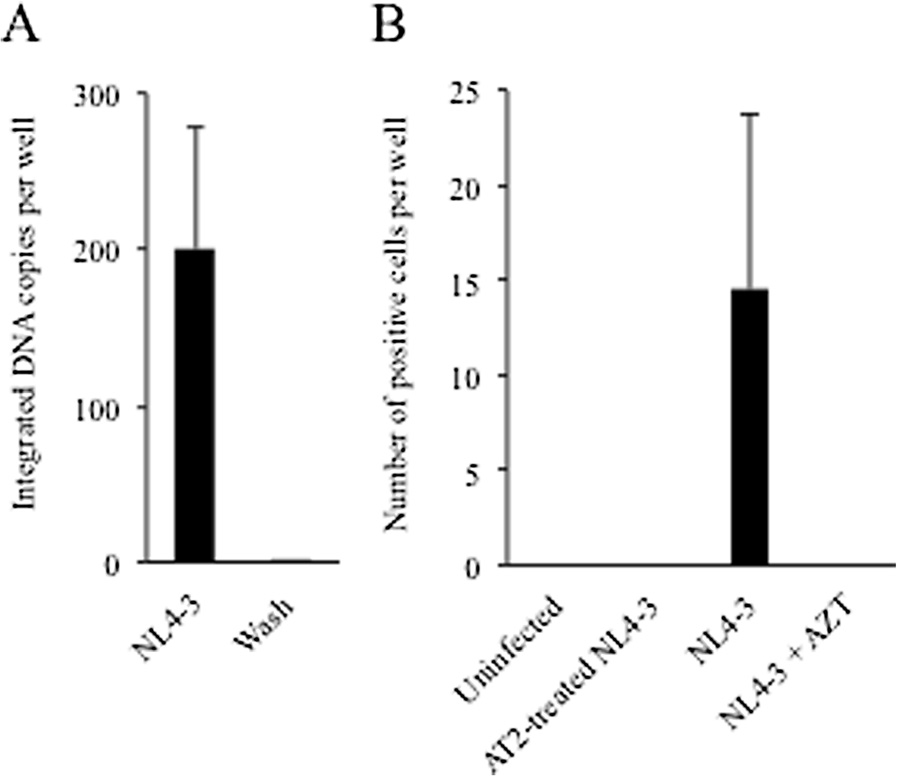
**HIV infection of primary hepatocytes.** (**A**) Primary human hepatocytes were incubated with DNase-treated HIV_NL4-3_ for two hours. After 24 hours, integrated HIV DNA was quantified in cell lysates by nested real-time PCR. (**B**)TZM-bl indicator cells were incubated with culture supernatants collected at day 3 post-infection from primary human hepatocytes infected with HIV_NL4-3_ or 100 ng AT-2 treated HIV_NL4-3_ and tested for β-galactosidase activity. The mean number of positive TZM-bl cells per well is shown with the error bars representing the standard deviation between duplicates. β-gal expression was also evaluated in the presence of 100 uM AZT for one hour prior to and during infection.

Because liver biopsies are not routinely performed in HIV mono-infected persons in the absence of viral hepatitis, there are limited data on HIV infection of hepatocytes *in vivo*. Moreover, mechanical dissociation of explant livers into their component cell types is challenging due to the infrequency of liver transplantation in HIV-positive indivixduals and the frequent cirrhosis that is found in these patients. Therefore, we further examined whether HIV could infect primary hepatocytes *in vitro*. Similar to the Huh7.5 and Huh7.5_JFH1_ cell lines, integrated HIV DNA was detected in primary hepatocytes after infection with HIV_NL4-3_ (Figure 4A). Incubation of supernatants from HIV-infected primary hepatocytes with the TZM-bl indicator cell line also showed modest numbers of positive cells (21 ± 14.5; Figure 4B). No TZM-bl positive cells were observed using supernatants from primary hepatocytes that were mock infected or exposed to AT-2-treated HIV_NL4-3_.

## Conclusions

Several lines of evidence suggest the potential for HIV to interact directly with multiple liver cell populations *in vivo* (reviewed in [9]). For instance, HIV RNA and proviral DNA have been detected in liver biopsies from persons with HIV infection [28-30]. Subsequent immunohistochemistry and *in situ* hybridization studies using liver specimens from HIV-infected patients have demonstrated HIV p24 protein and HIV RNA in Kupffer cells, inflammatory mononuclear cells, sinusoidal cells, intrahepatic lymphocytes, and/or hepatocytes [28,31-35]. Efficient activation of the HIV long terminal repeat also occurs in hepatocytes [36-38]. HIV compartmentalization in the liver has also been reported recently, suggesting that viral adaptation for replication within the liver may occur *in vivo *[30]. Soluble HIV proteins, such as gp120, also induce caspase expression and hepatic apoptosis [39-41], as well as interleukin 8 (IL-8), a pro-inflammatory chemokine that is an important mediator of hepatic inflammation and is known to antagonize the antiviral effects of IFN [42-45].

Despite multiple studies assessing the effects of HIV proteins on hepatocytes *in vitro*, contradictory reports of HIV entry receptor expression [39,40,46,47] may have lessened enthusiasm for investigating the ability of HIV to infect and replicate within hepatocytes. Nonetheless, several recent reports provide evidence that HIV can productively infect hepatocytes at low levels. For example, Xiao *et al.* isolated a CD4-independent strain of HIV from a patient with advanced HIV disease that was capable of infecting hepatocyte cell lines, as well as primary hepatocytes [48]. Fromentin *et al.* demonstrated that Huh7.5 cells bind to and internalize HIV particles and that HIV infection of CD4^+^ T cells was enhanced after interacting with virus-loaded hepatocytes compared to cell-free virus [49]. Finally, Iser *et al.* observed increased HIV reverse transcriptase activity following HIV infection of hepatocyte cell lines [50]. While the precise receptors used by HIV to enter hepatocytes remain elusive, Iser *et al.* reported that, despite the inability to detect surface expression of CD4, CCR5, or CXCR4 by flow cytometry, infection with R5 or X4 HIV was inhibited by maraviroc or AMD3100, respectively, suggesting CCR5- or CXCR4-dependent entry [50]. We have also detected CCR1, CCR2, and CCR3 co-receptors on Huh7.5 cells (Cardona-Maya and Blackard, unpublished data). Therefore, these receptors may also contribute to HIV infection of hepatocytes, although this requires additional investigation.

The current investigation was considered a proof-of-principle study designed to assess whether HIV was capable of infecting hepatocytes or not. Thus, several cell lines (HCV infected and HCV uninfected), as well as primary hepatocytes, were utilized. This study complements previous investigations and provides several novel findings. First, the Huh7.5 and Huh7.5_JFH1_ cell lines, as well as primary hepatocytes, supported early steps in the HIV life cycle as demonstrated by detection of integrated HIV DNA. Second, hepatocyte cell lines and primary hepatocytes also support late steps in the HIV life cycle as demonstrated by p24 detection in the supernatants and/or lysates of infected cells and infection of an HIV indicator cell line with hepatocyte-derived HIV. This finding is particularly interesting in light of the recent study by Fromentin *et al.* demonstrating that circulating CD4+ T cells can be potentially infected with HIV through contact with hepatocytes [49]. Our data further suggest that replication of HIV in hepatocytes is occurring at relatively low levels as integrated DNA levels in hepatocytes were lower than levels in Jurkats. Similarly, supernatants from HIV-infected hepatocytes contained fewer HIV particles that were able to infect an indicator cell line than HIV-infected Jurkats. These findings are in agreement with other recent reports that hepatocytes permit low-level replication of HIV [49,50].The levels of HIV infection achieved in primary hepatocytes versus hepatocyte cell lines were not equivalent. This may reflect differing levels of permissiveness to HIV in primary hepatocytes versus hepatocyte cell lines, possibly due to different levels of entry receptors or innate antiviral defense molecules. Interestingly, single-cell cloning has been utilized to examine permissiveness of Huh7 cells to HCV infection [51]. The finding of sub-populations of the parental cell line with distinct phenotypic characteristics implies that HIV infectivity could also be impacted, although a comprehensive analysis of HIV infection of primary hepatocytes derived from distinct patients has not been performed to date. Thus, future studies that address the expression of HIV entry receptors, as well as cellular factors that restrict HIV infection should be examined in primary hepatocytes from multiple donors and correlated with overall levels of HIV replication.

We were quite interested to find that HIV could indeed infect an HCV-infected hepatocyte cell line. In a preliminary investigation, we found that HIV infection of Huh7.5_JFH1_ cells resulted in increased positive- and negative-sense HCV RNA levels, as well as increased HCV protein expression, compared to HIV uninfected cells [52]. However, HIV-induced HCV replication was abolished in the presence of antiretroviral agents. These data complement *in vivo* reports that HCV RNA levels are elevated in individuals with HIV/HCV co-infection compared to those with HCV mono-infection [11,53,54] and suggest that the HCV-infected Huh7.5_JFH1_ cell line could be developed as an *in vitro* model to characterize mechanisms by which HIV and HCV interact at the cellular level and contribute to accelerated liver disease.

In conclusion, the present results demonstrate that the Huh7.5 and Huh7.5_JFH1_ cell lines, as well as primary hepatocytes, can be infected with HIV. These studies provide the necessary systems to further expand our understanding of virus-virus and virus-host interactions that are relevant for enhancing our understanding of how HIV impacts liver disease, as well as increased replication of hepatitis viruses. Moreover, these investigations could ultimately lead to optimization of current therapies for HIV and ameliorate the deleterious effects of HIV on liver disease.

## Competing interests

The authors declare that they have no competing interests.

## Authors’ contributions

LK participated in the study design and carried out virologic studies. WCM participated in the study design, carried out virologic studies, and helped draft the manuscript. MMF and GM assisted with virologic studies. MTS, KES, and CC participated in study design and coordination and assisted with manuscript editing. JTB designed and coordinated the study and drafted and edited the manuscript. All authors have read and approved the final manuscript.

## References

[B1] Keaveny AP Karasik M Hepatobiliary and pancreatic infections in AIDS: Part one AIDS Patient Care and STDs 1998 12 5 347 357 10.1089/apc.1998.12.347 11361970

[B2] Kahn JO Walker B Acute human immunodeficiency virus type 1 infection N Engl J Med 1998 339 1 33 39 10.1056/NEJM199807023390107 9647878

[B3] Bonacini M Hepatobiliary complications in patients with human immunodeficiency virus infection Am J Med 1992 92 404 411 10.1016/0002-9343(92)90271-C 1558086

[B4] Ingiliz P Liver damage underlying unexplained transaminase elevation in human immunodeficiency virus-1 mono-infected patients on antiretroviral therapy Hepatology 2009 49 2 436 442 10.1002/hep.22665 19085967

[B5] Lefkowitch J Pathology of AIDS-related liver disease Dig Dis 1994 12 6 321 330 10.1159/000171468 7712615

[B6] Sterling R Impact of highly active antiretroviral therapy on the spectrum of liver disease in HCV-HIV coinfection Clin Gastroenterol Hepatol 2004 2 5 432 439 10.1016/S1542-3565(04)00129-6 15118983

[B7] Mata-Marin JA Correlation between HIV viral and aminotransferases as liver damage markers in HIV infected naive patients: a concordance cross-sectional study Virol J 2009 6 181 10.1186/1743-422X-6-181 19878552 PMC2777159

[B8] Blackard JT HIV mono-infection is associated with FIB-4 - a noninvasive index of liver fibrosis - in women Clin Infect Dis 2011 52 5 674 680 10.1093/cid/ciq199 21248367 PMC3106241

[B9] Blackard JT Sherman K HCV/ HIV co-infection: time to re-evaluate the role of HIV in the liver? Journal of Viral Hepatitis 2008 15 5 323 330 10.1111/j.1365-2893.2008.00970.x 18208497 PMC3526832

[B10] Alter M Epidemiology of hepatitis C virus infection World J Gastroenterol 2007 13 17 2436 2441 17552026 10.3748/wjg.v13.i17.2436 PMC4146761

[B11] Tedaldi E Influence of coinfection with hepatitis C virus on morbidity and mortality due to human immunodeficiency virus infection in the era of highly active antiretroviral therapy Clin Infect Dis 2003 36 363 367 10.1086/345953 12539079

[B12] Bica I Increasing mortality due to end-stage liver disease in patients with human immunodeficiency virus infection Clin Infect Dis 2001 32 492 497 10.1086/318501 11170959

[B13] Salmon-Ceron D Liver disease as a major cause of death among HIV infected patients: role of hepatitis C and B viruses and alcohol Journal of Hepatolology 2005 42 6 799 805 10.1016/j.jhep.2005.01.022 15973779

[B14] Operskalski EA Kovacs A HIV/HCV co-infection: pathogenesis, clinical complications, treatment, and new therapeutic technologies Current HIV/AIDS Reports 2011 8 1 12 22 10.1007/s11904-010-0071-3 21221855 PMC3035774

[B15] Wakita T Production of infectious hepatitis C virus in tissue culture from a cloned viral genome Nature Medicine 2005 11 7 791 796 10.1038/nm1268 PMC2918402 15951748

[B16] Zhong J Robust hepatitis C virus infection in vitro Proc Natl Acad Sci 2005 102 26 9294 9299 10.1073/pnas.0503596102 15939869 PMC1166622

[B17] Heller T An in vitro model of hepatitis C virion production Proc Natl Acad Sci 2005 102 7 2579 2583 10.1073/pnas.0409666102 15701697 PMC549006

[B18] Lindenbach B Complete replication of hepatitis C virus in cell culture Science 2005 309 5734 623 626 10.1126/science.1114016 15947137

[B19] Blight KJ McKeating JA Rice C Highly permissive cell lines for subgenomic and genomic hepatitis C virus RNA replication J Virol 2002 76 24 13001 13014 10.1128/JVI.76.24.13001-13014.2002 12438626 PMC136668

[B20] Cai Z Robust production of infectious hepatitis C virus (HCV) from stably HCV cDNA-transfected human hepatoma cells J Virol 2005 79 22 13963 13973 10.1128/JVI.79.22.13963-13973.2005 16254332 PMC1280219

[B21] Platt EJ Effects of CCR5 and CD4 cell surface concentrations on infections by macrophage tropic isolates of human immunodeficiency virus type 1 J Virol 1998 72 4 2855 2864 9525605 10.1128/jvi.72.4.2855-2864.1998 PMC109730

[B22] Adachi A Production of acquired immunodeficiency syndrome-associated retrovirus in human and nonhuman cells transfected with an infectious molecular clone J Virol 1986 59 2 284 291 3016298 10.1128/jvi.59.2.284-291.1986 PMC253077

[B23] Haltiner M Kempe T Tjian R A novel strategy for constructing clustered point mutations Nucleic Acids Res 1985 13 3 1015 1025 10.1093/nar/13.3.1015 2987803 PMC341049

[B24] Rossio JL Inactivation of human immunodeficiency virus type 1 infectivity with preservation of conformational and functional integrity of virion surface proteins J Virol 1998 72 10 7992 8001 9733838 10.1128/jvi.72.10.7992-8001.1998 PMC110135

[B25] Kimpton J Emerman M Detection of replication-competent and pseudotyped human immunodeficiency virus with a sensitive cell line on the basis of activation of an integrated beta-galactosidase gene J Virol 1992 66 4 2232 2239 1548759 10.1128/jvi.66.4.2232-2239.1992 PMC289016

[B26] Chomont N HIV reservoir size and persistence are driven by T cell survival and homeostatic proliferation Nature Medicine 2009 15 8 893 900 10.1038/nm.1972 PMC2859814 19543283

[B27] Moreno-Fernandez ME Human regulatory T cells are targets for human immunodeficiency virus (HIV) infection, and their susceptibility differs depending on the HIV type 1 strain J Virol 2009 83 24 12925 12933 10.1128/JVI.01352-09 19828616 PMC2786841

[B28] Cao YZ Identification and quantitation of HIV-1 in the liver of patients with AIDS AIDS 1992 6 1 65 70 10.1097/00002030-199201000-00008 1543567

[B29] Donaldson YK Redistribution of HIV outside the lymphoid system with onset of AIDS Lancet 1994 343 8894 383 385 7905551 10.1016/s0140-6736(94)91222-x

[B30] Blackard JT HIV variability in the liver and evidence of possible compartmentalization AIDS Research and Human Retroviruses 2011 27 10 1117 1126 10.1089/aid.2010.0329 21417757 PMC3186706

[B31] Housset C Lamas E Brechot C Detection of HIV1 RNA and p24 antigen in HIV-1-infected human liver Res Virol 1990 141 2 153 159 10.1016/0923-2516(90)90017-D 1971728

[B32] Housset C Presence of HIV-1 in human parenchymal and non-parenchymal liver cells in vivo J Hepatol 1993 19 2 252 256 10.1016/S0168-8278(05)80579-3 8301058

[B33] Hoda SA White JE Gerber M Immunohistochemical studies of human immunodeficiency virus-1 in liver tissues of patients with AIDS Mod Pathol 1991 4 5 578 581 1758870

[B34] Lang ZW A pathological study on liver tissues of patients with HIV infection Zhonghua Gan Zang Bing Za Zhi 2005 13 12 930 932 16381643

[B35] Jiang TJ Immunohistochemical evidence for HIV-1 infection in the liver of HIV-infected patients Zhonghua Shi Yan He Lin Chuang Bing Du Xue Za Zhi 2005 19 2 152 154 16027784

[B36] Zhu M Duan L Pomerantz R TAR- and Tat-independent replication of human immunodeficiency virus type 1 in human hepatoma cells AIDS Research and Human Retroviruses 1996 12 12 1093 1101 10.1089/aid.1996.12.1093 8844014

[B37] Pizzella T Banerjee R Identification of a human immunodeficiency virus type 1 TAR binding protein in human hepatoblastoma HepG2 cells that trans-activates HIV-1 LTR-directed gene expression DNA Cell Biology 1994 13 1 67 74 10.1089/dna.1994.13.67 8286041

[B38] Hsu ML Cytokine regulation of HIV-1 LTR transactivation in human hepatocellular carcinoma cell lines Cancer Lett 1995 94 1 41 48 10.1016/0304-3835(95)03820-M 7621443

[B39] Vlahakis S Human immunodeficiency virus-induced apoptosis of human hepatocytes via CXCR4 J Infect Dis 2003 188 1455 1460 10.1086/379738 14624370

[B40] Munshi N Hepatitis C and human immunodeficiency virus envelope proteins cooperatively induce hepatocytic apoptosis via an innocent bystander mechanism J Infect Dis 2003 188 1192 1204 10.1086/378643 14551890

[B41] Balasubramanian A Molecular mechanism of hepatic injury in coinfection with hepatitis C virus and HIV Clin Infect Dis 2005 41 Suppl 1 S32 S37 16265611 10.1086/429493

[B42] Balasubramanian A Ganju R Groopman J HCV and HIV envelope proteins collaboratively mediate IL-8 secretion through activation of p38 MAP kinase and SHP2 in hepatocytes J Biol Chem 2003 278 37 35755 35766 10.1074/jbc.M302889200 12824191

[B43] Polyak S Elevated levels of interleukin-8 in serum are associated with hepatitis C infection and resistance to interferon therapy J Virol 2001 75 13 6209 6211 10.1128/JVI.75.13.6209-6211.2001 11390624 PMC114338

[B44] Polyak S Hepatitis C virus nonstructural 5A protein induces interleukin-8, leading to partial inhibition of the interferon-induced antiviral response J Virol 2001 75 13 6095 6106 10.1128/JVI.75.13.6095-6106.2001 11390611 PMC114325

[B45] Khabar K The alpha chemokine, interleukin 8, inhibits the antiviral action of interferon a Journal of Experimental Medicine 1997 186 7 1077 1085 10.1084/jem.186.7.1077 9314556 PMC2199072

[B46] Cao YZ CD4-independent, productive human immunodeficiency virus type 1 infection of hepatoma cell lines in vitro J Virol 1990 64 6 2553 2559 2159530 10.1128/jvi.64.6.2553-2559.1990 PMC249431

[B47] Banerjee R Inhibition of HIV-1 productive infection in hepatoblastoma HepG2 cells by recombinant tumor necrosis factor-a AIDS 1992 6 10 1127 1131 10.1097/00002030-199210000-00010 1466843

[B48] Xiao P Characterization of a CD4-independent clinical HIV-1 that can efficiently infect human hepatocytes through chemokine (C-X-C motif) receptor 4 AIDS 2008 22 14 1749 1757 10.1097/QAD.0b013e328308937c 18753859

[B49] Fromentin R MR T Tremblay M Human hepatoma cells transmit surface bound HIV-1 to CD4+ T cells through an ICAM-1/LFA-1-dependent mechanism Virology 2010 398 168 175 10.1016/j.virol.2009.12.008 20034651

[B50] Iser DM Coinfection of hepatic cell lines with human immunodeficiency virus and hepatitis B virus leads to an increase in intracellular hepatitis B surface antigen J Virol 2010 84 12 5860 5867 10.1128/JVI.02594-09 20357083 PMC2876638

[B51] Akazawa D CD81 expression is important for the permissiveness of Huh7 cell clones for heterogeneous hepatitis C virus infection J Virol 2007 81 10 5036 5045 10.1128/JVI.01573-06 17329343 PMC1900197

[B52] Kong L Welge JA Blackard J Infectious HIV increases hepatitis C virus (HCV) expression in hepatocytes18th Conference on Retroviruses and Opportunistic Infections 2011 MA: Boston

[B53] Yokozaki S Immunological dynamics in hemophiliac patients infected with hepatitis C and human immunodeficiency virus: influence of antiretroviral therapy Blood 2000 96 13 4293 4299 11110704

[B54] Beld M Evidence that both HIV and HIV-induced immunodeficiency enhance HCV replication among HCV seroconverters Virology 1998 244 504 512 10.1006/viro.1998.9130 9601518

